# Real-World Outcomes of Chemiluminescence Immunoassay Implementation for *Mycobacterium tuberculosis* Infection Screening

**DOI:** 10.1093/ofid/ofag234

**Published:** 2026-04-22

**Authors:** Eli Wilber, Marcos C Schechter, Paulina A Rebolledo, Sheetal Kandiah, Bruce Aldred, Yun F Wang, Susan M Ray

**Affiliations:** Division of Infectious Diseases, Department of Medicine, Emory University School of Medicine, Atlanta, Georgia, USA; Grady Health System, Atlanta, Georgia, USA; Division of Infectious Diseases, Department of Medicine, Emory University School of Medicine, Atlanta, Georgia, USA; Grady Health System, Atlanta, Georgia, USA; Division of Infectious Diseases, Department of Medicine, Emory University School of Medicine, Atlanta, Georgia, USA; Grady Health System, Atlanta, Georgia, USA; Hubert Department of Global Health, Rollins School of Public Health, Emory University, Atlanta, Georgia, USA; Division of Infectious Diseases, Department of Medicine, Emory University School of Medicine, Atlanta, Georgia, USA; Grady Health System, Atlanta, Georgia, USA; Division of Infectious Diseases, Department of Medicine, Emory University School of Medicine, Atlanta, Georgia, USA; Grady Health System, Atlanta, Georgia, USA; Grady Health System, Atlanta, Georgia, USA; Department of Pathology and Laboratory Medicine, Emory University School of Medicine, Atlanta, Georgia, USA; Division of Infectious Diseases, Department of Medicine, Emory University School of Medicine, Atlanta, Georgia, USA; Grady Health System, Atlanta, Georgia, USA

## Abstract

Fully automated interferon gamma release assays based on chemiluminescence immunoassay promise more efficient testing for tuberculosis infection but increase rates of positive test results when compared with semiautomated assays. We report an increase in borderline-positive interferon gamma release assay results (87.8% relative increase; 95% CI, 31.1%–170.0%), which led to modifications in clinical care following chemiluminescence immunoassay implementation.

Interferon gamma release assays (IGRAs) are often the preferred method of screening for tuberculosis (TB) infection due to the need for only a single clinical encounter and increased test specificity when testing individuals vaccinated with bacillus Calmette-Guérin [1]. High-volume TB infection screening can place strains on laboratory workflows due to the associated labor and technical requirements of the enzyme-linked immunosorbent assays (ELISAs) used to quantify the interferon gamma [2]. A fully automatic chemiluminescence immunoassay (CLIA) recently became available in the United States with the promise of improving laboratory workflow [2]. Initial evaluations of this assay have suggested low repeatability and increased positive results relative to the prior standard of ELISA [3–8]. Increased interferon gamma values with CLIA relative to ELISA have also been observed when measuring a standardized curve of contrived specimens [3]. The downstream clinical impacts of these changes in performance characteristics have not been well studied. We aimed to characterize the clinical impact of CLIA implementation on our health system and the potential need for new diagnostic stewardship measures.

## METHODS

This study was conducted at Grady Health System in Atlanta, Georgia, which serves Fulton and Dekalb counties (combined population ∼1.8 million persons) [9]. Grady Health System consists of a single large academic hospital (953 licensed beds, >260 000 patient days in 2025) with medical, surgical, and specialty clinics, as well as a large HIV clinic and neighborhood clinics. All employees are screened for TB infection at the time of employment. In 2025 there were 26 new diagnoses of TB across Grady Health System.

We conducted a retrospective chart review of all IGRAs conducted at Grady Health System from 1 January 2021 to 31 March 2025, including employee health and patients. The ELISA-based testing strategy—QuantiFERON-TB Gold Plus by Qiagen on semiautomated DSX by Dynex—was in use for the period of 1 January 2021 to 12 May 2023. The fully automated CLIA testing approach—LIAISON QuantiFERON-TB Gold Plus on LIAISON XL by DiaSorin—was implemented on 13 May 2023. We compared the rate of test positivity (TB1–nil or TB2–nil ≥0.34), borderline positivity (TB1–nil or TB2–nil 0.34≤ × <1.0 and neither ≥1.0), and nonborderline positivity (TB1–nil or TB2–nil ≥1.0) after CLIA implementation to the historical rate using the 2-proportion *z* test. An interrupted time series (ITS) analysis based on a quasi-Poisson regression model (with a level-change parameter) was used to analyze the testing change. For patients with multiple tests (employee health data excluded), we calculated the rate of seroconversion and conducted chart review to assess any impact on management. Statistical analysis was conducted in RStudio with figures created in the package “ggplot2.” This analysis was conducted as a quality improvement project to inform diagnostic stewardship.

## RESULTS

**Figure 1.**
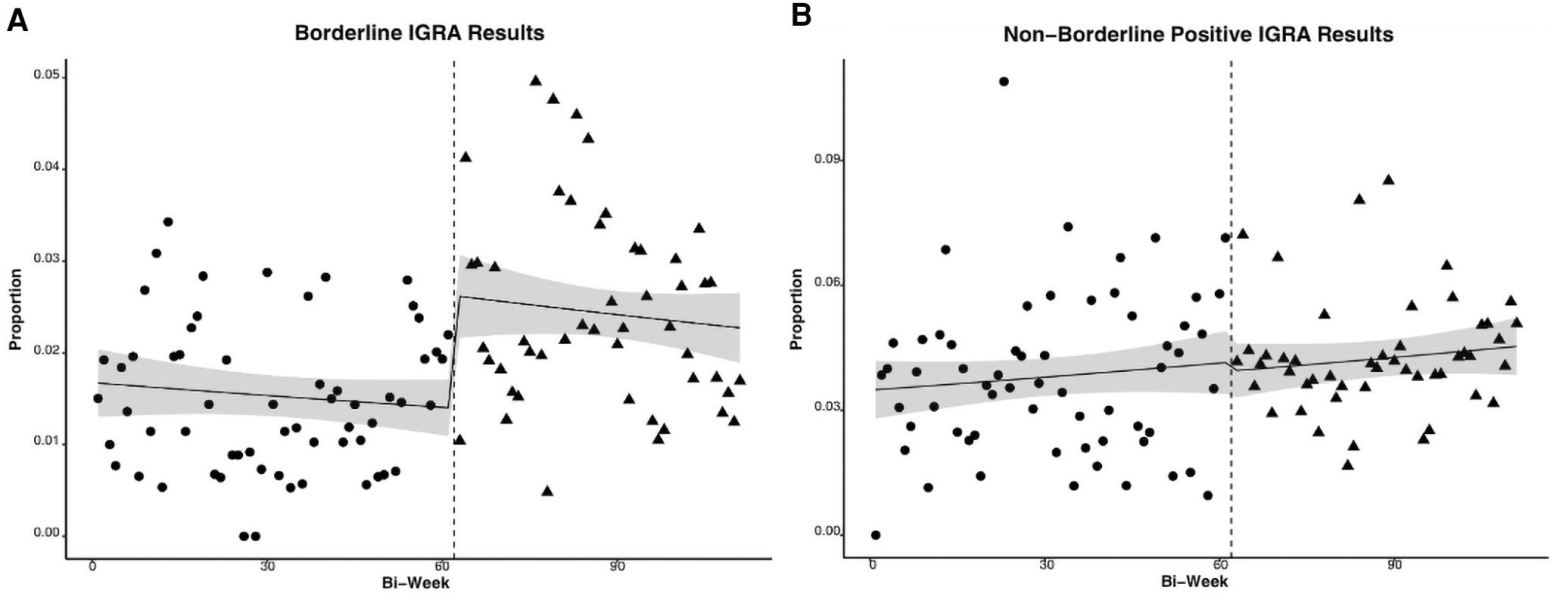
Interrupted time series of biweekly rates of positive IGRA results: *A*, borderline; *B*, nonborderline. Circles/triangles: pre- and postintervention observed rates. Line: quasi-Poisson regression model with 95% CI (shaded). IGRA = interferon gamma release assay.

In total, 22 392 IGRAs were performed during the study period (Table 1). Rates of positivity (6.7% vs 5.4%, *P* < .001) and borderline positivity (2.5% vs 1.6%, *P* < .001) were higher for CLIA than ELISA. Nonborderline-positive results were not significantly different. In ITS analysis (Figure 1), CLIA implementation was associated with a significant level change in the borderline positivity rate (87.8% relative increase; 95% CI, 31.1%–170.0%) but not the nonborderline positivity rate (6.2% relative decrease; 95% CI, −31.9 to +30.4%).

**Table 1.** Distribution of Interferon Gamma Release Assay Results

	ELISA (n = 10 248)		CLIA (n = 12 144)		
	No.	%	No.	%	*P* Value
All tests					
Positive	552	5.4	813	6.7	<.001
Nonborderline	393	3.8	515	4.2	.134
Borderline	159	1.6	298	2.5	<.001

	ELISA (n = 545)		CLIA (n = 1383)		
Repeat tests					
Positive	2	0.4	27	2.0	.018
Nonborderline	1	0.2	5	0.4	.859
Borderline	1	0.2	22	1.6	.02

Abbreviations: CLIA, chemiluminescence immunoassay; ELISA, enzyme-linked immunosorbent assay.

Of 1928 instances of repeat IGRA testing on clinical patients (>2 test results during the study period), there were 29 seroconversions (1.5%). In 27 instances, the newly positive result was a borderline-positive result. Repeat tests on the CLIA were more likely to be a seroconversion (2.0% vs 0.4%, *P* = .018). On chart review, 15 of 27 borderline seroconversions were referred for TB infection treatment, and 8 of 27 had a therapy modification (eg, disease-modifying antirheumatic drug interruption).

## DISCUSSION

IGRAs are an important tool for TB infection screening given their logistical advantages over tuberculin skin tests and the lack of other diagnostic options for detecting TB infection. The new CLIA-based IGRA is an attractive option for diagnostic laboratories given the high-throughput automated processing [2]. However, multiple analyses conducted by different investigators have consistently demonstrated a signal of higher interferon gamma values and a resultant increase in positive test results when using this test platform vs the historical ELISA standard [3–8]. TB infection diagnosis is challenging and requires the evaluation of multiple factors, and it is possible that the increased test positivity with CLIA represents an increase in true infections. Yet, the majority of these studies (and our own) have been conducted in low-incidence settings, increasing the likelihood that these additional positive results reflect false positives (ie, patients who do not have TB infection). We replicate these findings in real-world clinical data and demonstrate adverse impacts on the care of some patients.

Our analysis uses a robust statistical tool (ITS) to demonstrate the impact of ELISA to CLIA switching on the proportion of borderline-positive IGRA results in a nonendemic setting. Retrospective chart review revealed that over half of patients with a “seroconversion” after testing methodology was switched to CLIA were referred for TB infection treatment. Approximately one-half of those referred for treatment experienced an interruption in therapy for the underlying disease state (inflammatory bowel disease, HIV, etc) that prompted repeat TB infection screening. Anecdotally, some of the patients arrive to the infectious diseases clinic highly symptomatic from relapse or flaring of their underlying disease that was previously well controlled by immunosuppressive medications that had been discontinued by their primary providers.

Other centers have implemented diagnostic stewardship measures involving repeat testing of borderline-positive results to mitigate the impact of these likely false-positive results, with a resultant significant decrease in the number of positive results reported [10]. Whether this is the best approach for incorporating the CLIA into routine use remains to be determined and could vary by institutional resources and the population being tested. At present, we have opted for direct engagement with stakeholders in referring clinics to expedite expert evaluation of high-risk cases with positive IGRAs to minimize/prevent disruptions in disease-modifying therapies. Leveraging the electronic health record to provide clinical decision support at the time of test ordering and/or resulting may be one method to make this approach more reliable while incorporating the principles of diagnostic stewardship [11]. Clinical decision support can be used to improve diagnostic test selection to decrease testing in low pretest situations (eg, annual testing without new exposures); however, we have found anecdotally that annual testing is often driven by the requirements or perceived requirements of prior authorization processes for immunosuppressive agents. Prior research has demonstrated the shortcomings of IGRAs when used for serial testing in settings with low TB incidence [12, 13] and efforts to minimize this practice when able are worthwhile. Additionally, clinical decision support can be used when results are viewed by the ordering provider to guide interpretation and recommend expert consultation.

Our study is limited by being a single-center investigation and the lack of a true gold standard for determining TB infection status. However, our findings are consistent with those of other investigators [4–8], which suggest that these findings are generalizable across low-incidence settings. Additionally, the ITS methodology is a robust quasi-experimental design [14] that, in this case, allows for a high degree of confidence in the conclusion that a shift to CLIA-based testing is the cause of the increase in borderline-positive IGRA results that are like false positives. The ability to review clinical data and demonstrate real-world adverse impacts of the CLIA-based testing's increased positivity rate is a new finding that extends this work beyond what has previously been reported. Institutions in low-incidence settings currently using or considering adopting CLIA-based testing for TB infection testing should be aware of the performance limitations of this assay and the potential clinical impacts of these limitations. Future work should focus on what diagnostic stewardship methodologies are best able to mitigate these limitations and allow for the improvements in laboratory efficiency without sacrificing high-quality patient care. Considerations on how to interpret IGRA results based on the methodology used for quantification (in conjunction with pretest probability and other clinical factors) may be necessary in future guideline documents (eg, World Health Organization, Centers for Disease Control and Prevention).
